# Fine Mapping and Candidate Gene Analysis of *BnC08.cds*, a Recessive Gene Responsible for Sepal-Specific Chlorophyll-Deficiency in *Brassica napus* L.

**DOI:** 10.3389/fpls.2022.850330

**Published:** 2022-03-10

**Authors:** Wei Zhang, Xiaoying Zhou, Feng Chen, Hongli Zhu, Rui Shi, Chengming Sun, Song Chen, Maolong Hu, Jiefu Zhang, Xiaodong Wang

**Affiliations:** ^1^Key Laboratory of Jiangsu Province for Agrobiology, Institute of Industrial Crops, Jiangsu Academy of Agricultural Sciences, Nanjing, China; ^2^State Key Laboratory of Crop Genetics and Germplasm Enhancement, Nanjing Agricultural University, Nanjing, China

**Keywords:** *Brassica napus* L, chlorophyll-deficient mutant, fine-mapping, RNA-sequencing, yellow-sepal, candidate gene

## Abstract

Chloroplast development is crucial for photosynthesis and plant growth and many factors are involved in its regulation. The regulatory mechanism differs in different green tissues, and previous studies have focused on chloroplasts in leaves. In this study, a mutant with sepal-specific chlorophyll-deficiency was observed in *Brassica napus* and named as df74. Genetic analysis indicated that the phenotype was controlled by a single recessive nuclear gene. The gene was located on chromosome C08 by bulked-segregant analysis with whole-genome sequencing, which was designated as *BnC08.cds*. To fine-map the *BnC08.cds*, a F_2_ population was created from the cross of df74 and Zhongshuang11 (ZS11). Finally, the *BnC08.cds* was fine-mapped in the region between the single-nucleotide polymorphism (SNP) markers M5 and M6, corresponding to a 228.72 kb interval of the *B. napus* “ZS11” genome. Eighteen genes were predicted in the target region, and it was speculated that *BnaC08G0442100ZS* was the most likely candidate gene based on the results of transcriptome analyses and sequence variation analyses. These results provide a foundation to explore the regulation of chloroplast development in sepals.

## Introduction

Green tissues are the photosynthetic organs in plants and their color is affected by the chlorophyll (Chl) content, which is the key factor for photomorphogenesis and photosynthesis. The biosynthesis and accumulation of Chl is a complex physiological and biochemical process. The mutation of any gene in this process may affect the content of Chl, so color mutants can readily form in artificial or spontaneous mutagenesis. The mutants are diverse, such as etiolation (Zhu et al., 2014), albino (Wang et al., 2016), white to green (Zhao et al., 2013; Ma et al., 2017), and stay-green (Jiang et al., 2007). These mutants are important for the study of Chl biosynthesis and degradation, chloroplast development, and chloroplast-nuclear signal transmission (Fu et al., 2019).

According to previous research, the factors involved in Chl content can be roughly divided into three categories: biosynthesis and degradation of Chl, metabolism of heme, and chloroplast development. In the first category, biosynthesis of Chl involves 16 enzymes encoded by 28 genes in *Arabidopsis thaliana* (Tanaka and Tanaka, 2007). The mutation of genes in this pathway may hinder the synthesis of Chl and cause chlorosis. On the contrary, mutations in genes related to the pathway of Chl degradation often trigger stay-green (Thomas and Ougham, 2014). Genes involved in metabolism of heme belong to the second category. The biosynthesis of Chl and heme are two branches of the tetrapyrrole biosynthetic pathway. They share precursors from 5-aminolevulinic acid to protoporphyrin IX, and they inhibit each other. If the content of heme increases, the excess heme will inhibit the biosynthesis of Chl *via* feedback, resulting in reduced Chl which causes the color mutation (Brzezowski et al., 2015). The third category is genes involved in chloroplast development. This is a complex network including chloroplast and nuclear gene transcription, RNA modification and processing, protein translation, folding and transport, and thylakoid formation (Beck, 2001). Mutations in any one of the genes may cause the blocking of chloroplast development and lead to color mutation.

The biogenesis and development of chloroplasts differ between organs and species (Pogson and Albrecht, 2011). It is useful to understand the molecular mechanisms underlying tissue-specific chloroplast development. Genetic studies have demonstrated that chloroplast development proceeds differently in cotyledons and true leaves. The *var2* mutants produce green cotyledons but chlorotic true leaves (Liu et al., 2010). Conversely, the *snowy cotyledon* (*sco*) mutants have green true leaves but chlorotic or bleached cotyledons (Albrecht et al., 2006, 2008, 2010). Burley is another important tissue-specific chlorophyll-deficient mutant. It is a special type of tobacco (*Nicotiana tabacum* L.) cultivar that is characterized by white stem and leaf midvein. A two-step mutation process in the double *White Stem 1* homologs has driven the evolution of burley tobacco (Wu et al., 2020). A similar phenotype was identified from a tomato mutant with albinic stems and this is caused by a mutation of *SlARC6*, which leads to the tissue-specific defects in chloroplast development (Chang et al., 2021).

Sepal is a kind of green tissue in many higher plants. It plays an important role in protecting the other floral organs. Sepals are thought to resemble leaves morphologically because of their green color and Chl content. In this study, we obtained a rapeseed mutant (named df74) with Chl-deficient sepals by ethyl methane sulfonate (EMS) mutagenizing. The mutant produces pale yellow sepals while the color of other green tissues stays normal. This kind of sepal-specific Chl-deficiency, as far as we know, has rarely been reported and it is a good candidate for studying the regulation of chloroplast development in sepals. Our genetic analysis has revealed that the phenotype of df74 is controlled by a single recessive nuclear gene. The gene was mapped on chromosome C08, in an interval of 228.72 kb physical distance. The comparative transcriptomic analysis showed that three differentially expressed genes (DEGs), which encode proteins participate in Chl breakdown were upregulated in df74, and one DEG participating in Chl biosynthesis was downregulated. The results will promote the map-based cloning of the candidate gene as well as the understanding of the regulation of chloroplast development in sepals of *Brassica napus*.

## Materials and Methods

### Plant Materials and Growth Conditions

The rapeseed mutant (df74) with Chl-deficient sepals was obtained from the conventional variety Ningyou 18 (NY18), whose seeds were treated with 1% EMS for 12 h. The mutant with a pale-yellow sepal phenotype was identified and was selfed to generate an inbred line. The df74 is smaller and shorter than NY18. The investigation of the F2 individuals derived from the cross of df74 and NY18 showed that the dwarfism and sepal-specific chlorophyll-deficiency are not linked to each other. So, we focused on the sepal-specific chlorophyll-deficiency phenomenon in this study. The cross between df74 and NY18 was carried out, and the F_2_ population was derived from the self-pollination of F_1_ plants. The BC_1_ was derived by the backcrosses of F_1_ to df74. The phenotype of the reciprocal hybrid F_1_ and the segregation ratio of F_2_ and BC_1_ population were used to detect the genetic pattern of the Chl-deficient sepal mutant. Another conventional variety Zhongshuang11 (ZS11) with normal green sepals was used to cross with df74 to construct a F_2_ population for fine mapping the candidate gene. All the plants were grown in fields located in Jiangsu Academy of Agricultural Sciences, Nanjing, Jiangsu Province, China, under normal cultivation.

### Measurement of Photosynthetic Pigments and Transmission Electron Microscopy Analysis

For pigment extraction, 200 mg fresh weight of sepals were harvested from df74 and NY18, respectively, and then extracted with 5 ml 95% ethanol. Specific Chl contents were measured at wavelengths of 665 and 649 nm with UV-2450 UV/Vis Spectrophotometers (Shimadzu Corporation, Kyoto, Japan) according to the method of Lichtenthaler (1987). Each measurement involved three biological replicates.

For transmission electron microscopy (TEM) analysis, sections were manufactured as described by Wang et al. (2016). Sepals at the same stage on df74 and NY18 were fixed in 2.5% glutaraldehyde and further fixed in 1% osmic acid, dehydrated in gradient acetone, embedded in Epon812 and sectioned, and then double stained with 2% uranyl acetate and lead citrate. The sections were observed and photographed under a Hitachi H-7650 TEM (Hitachi, Tokyo, Japan).

### Bulked-Segregant Analysis With Whole-Genome Sequencing

Two contrasting DNA pools were constructed with equal amounts of DNA from 25 green-sepal plants (G-pool) and 25 yellow-sepal plants (Y-pool) from the F_2_ populations derived from df74 × NY18. The two bulks and two parents were sequenced and the data were generated using the Illumina HiSeq™ PE150 (Illumina, Inc.; San Diego, CA, United States) platform. The sequence depth of the parents were ×30 genome coverage and the two bulks × 45. Sequencing and data analysis were performed by Novogene Bioinformatics Technology Co. Ltd. (Beijing, China). The clean reads of each sample were aligned to the *B. napus* “Darmor-*bzh*” reference genome (Chalhoub et al., 2014) using BWA software (Li and Durbin, 2009), and multiple read pairs were removed by the SAMtools command “rmdup” (Li et al., 2009). Sequence variants including single-nucleotide polymorphisms (SNPs) and small insertions and deletions (INDELs) were called using the Unified Genotyper function in GATK software (McKenna et al., 2010). SNPs with read depth <8 or quality score in scale <20 were filtered out. Only the SNPs that were polymorphic between the parents and homozygous in either parent were selected for the further analysis. Using df74 as the reference parent, SNP-index of the two bulks were calculated and the corresponding SNP-index graphs were plotted to find the candidate regions responsible for phenotypic variation. The Δ(SNP-index) can be estimated by the formula: Δ(SNP-index) = SNP-index (G-pool) − SNP-index (Y-pool). The SNPs with significantly different Δ(SNP-index; *p* < 0.01) were considered closely linked to the causal gene (Yaobin et al., 2018).

### Fine Mapping of the Candidate Gene

A F_2_ population with 3,911 individuals was derived from the cross between df74 and ZS11. The yellow-sepal individuals were selected for genotyping. According to the results of the sequencing of df74 and NY18, the SNPs between the two parents flanking the candidate interval were screened. The penta-primer amplification refractory mutation system (PARMS) was used to screen recombinant plants for fine-mapping. The primers were designed as described by Lu et al. (2020). The master mix for PARMS markers was purchased from Gentides Biotech Co. Ltd. (Wuhan, China). The parents, F_1_, 10 yellow-sepal individuals, and 10 green-sepal individuals were used to detect the reliability of PARMS markers. Then, two polymorphic markers (M1 and M2) were used to screen the yellow-sepal individuals of the F_2_ population. The recombinant individuals were selected to conduct the next round of genotyping. New PARMS SNP markers (M3–M10) were designed step by step based on the physical map of ZS11 (Song et al., 2020) to narrow the mapping interval gradually.

### RNA Extraction and Sequencing

The sepals of NY18 and df74 were harvested and immediately frozen in liquid nitrogen and then stored at −80°C for RNA extraction and transcriptome analysis. Three biological replicates were performed. Total RNA was extracted using a MiniBEST Plant RNA Extraction Kit (TaKaRa, Dalian, China). RNA-sequencing (RNA-seq) was carried out by Novogene Bioinformatics Technology Co. Ltd. The RNA quality was determined by a Qubit RNA Assay Kit in Qubit 2.0 Fluorometer (Life Technologies, Carlsbad, CA, United States), RNA Nano 6000 Assay Kit, and Bioanalyzer 2100 system (Agilent Technologies, Santa Clara, CA, United States). Then, six sequencing libraries were constructed and sequenced on Illumina Hiseq 2000 platform, and 100 bp paired-end reads were generated. These methods were described by Wang et al. (2020).

### RNA-Seq Data Analysis

Raw RNA-seq reads were filtered by removing reads containing the adapter, and reads containing ploy-Ns and low-quality reads. Then, the clean reads were aligned to the *B. napus* “Darmor-*bzh*” reference genome using the hisat2 program (Wen, 2017). DESeq2 was used for filtering the DEGs with |logFC(log_2_fold change)| ≥ 1 and fdr < 0.05. The Gene Ontology (GO) annotation of DEGs was performed by the GOseq R package (Young et al., 2010), and GO terms with corrected *p* < 0.05 were considered as significantly enriched terms. The Kyoto Encyclopedia of Genes and Genomes (KEGG) pathway annotation was carried out with a BLAST search against the KEGG database.^1^ Pathway enrichment analysis was performed by KOBAS 2.0.^2^

## Results

### Phenotypic Characterization

**Figure 1:**
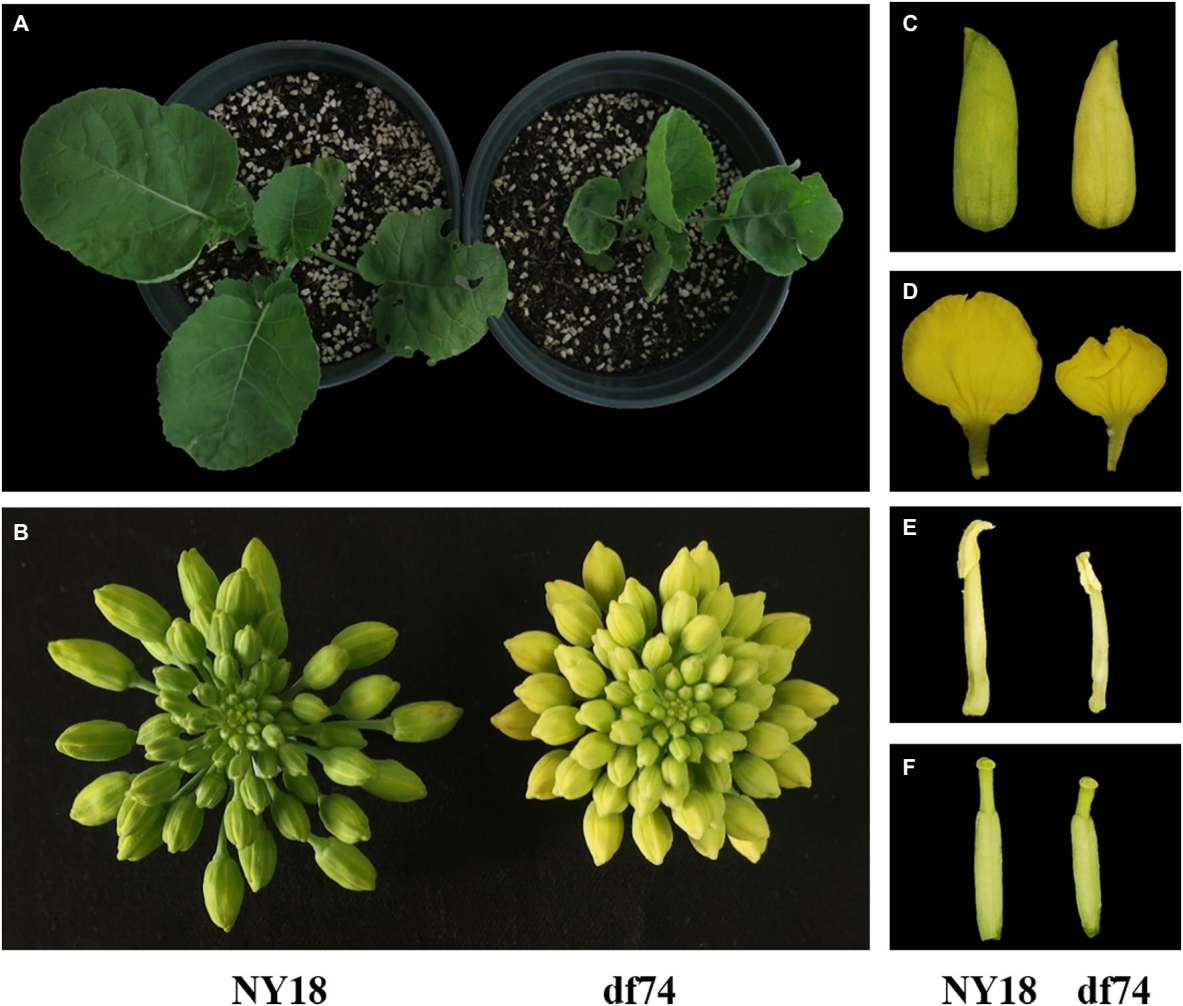
Phenotypic characterization of Ningyou 18 (NY18) and df74. **(A)** NY18 and df74 at seedling stage; **(B)** inflorescence of NY18 and df74; **(C–F)** sepal, petal, stamen, and carpel of NY18 and df74.

**Figure 2:**
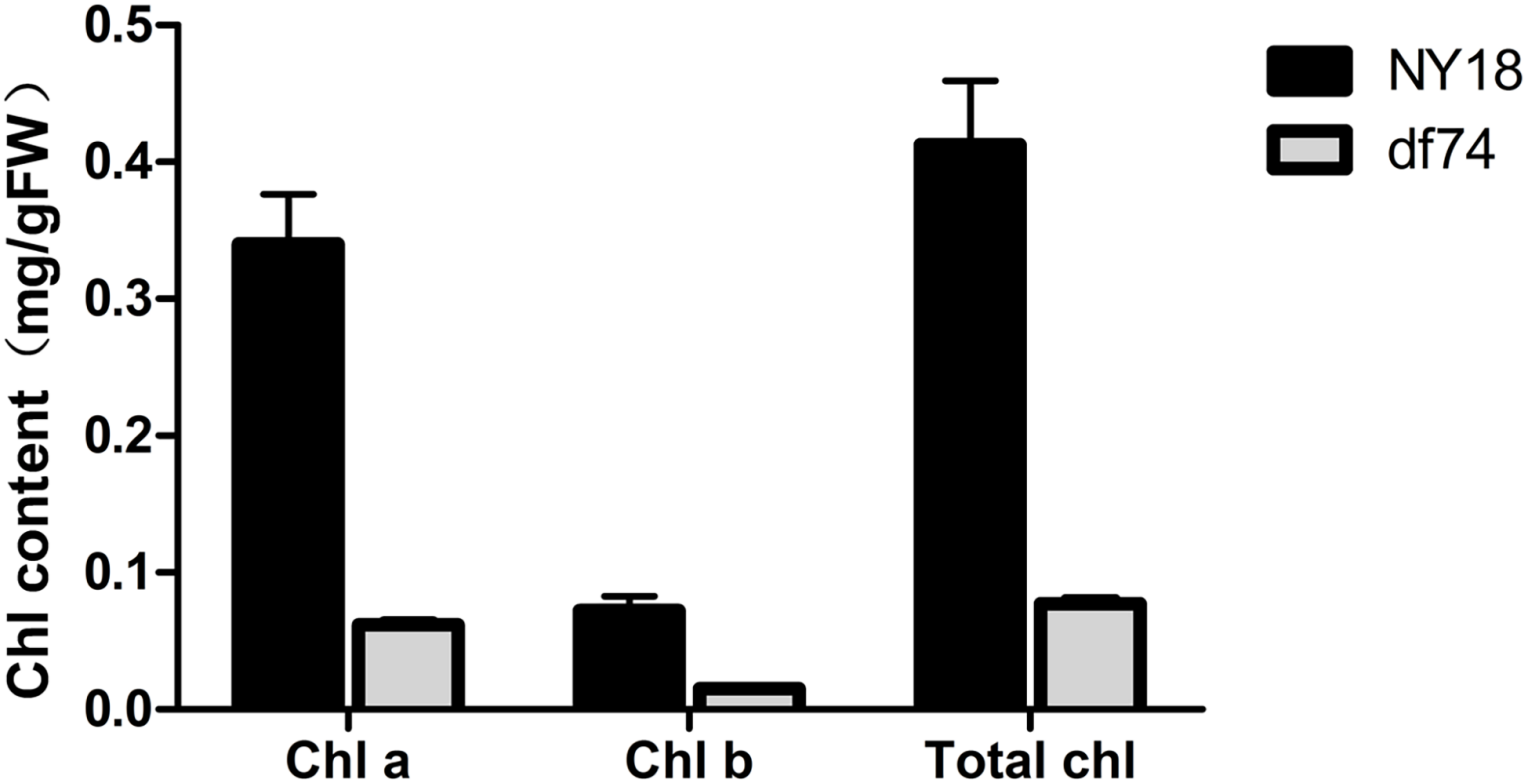
Chlorophyll (Chl) contents in sepals of NY18 and df74.

In the seedling stage, the leaves of df74 were as green as NY18, but smaller than NY18 (Figure 1A). The df74 entered the bolting stage and flowering stage 3–5 days later than NY18, and had smaller floral organs (Figures 1B–F). The sepals of df74 were pale yellow, but the other green tissues including leaves, stems, and carpels, had the same green color as NY18. The Chl content of sepals in df74 was significantly different from NY18. Sepals of df74 had 82, 71, and 80% reduction of Chl *a*, Chl *b*, and total Chl, respectively, compared with NY18 (Figure 2).

**Figure 3:**
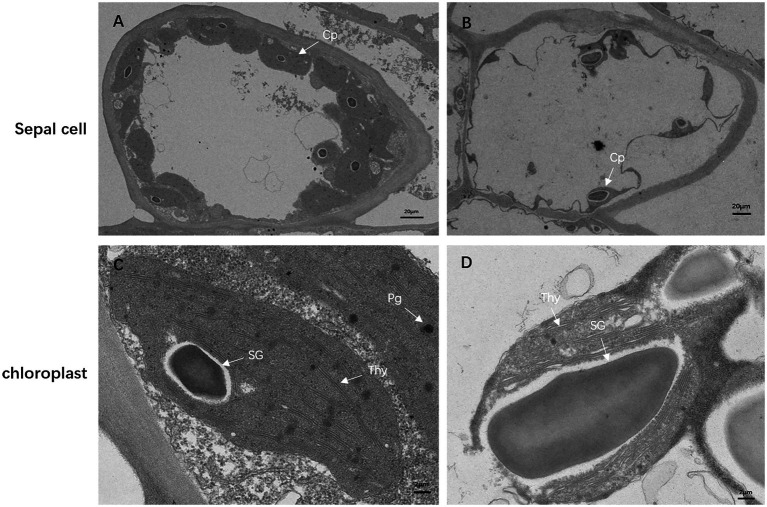
Transmission electron micrographs of chloroplasts from NY18 **(A,C)** and df74 **(B,D)**. Examples of chloroplast (Cp), plastoglobule (Pg), starch granules (SG), and thylakoid (Thy). Bars: 20 μm **(A,B)**, 2 μm **(C,D)**.

To investigate how the mutation affects chloroplast development, we observed the ultrastructure of the chloroplasts in the sepals of df74 and NY18 with TEM. In the cells of NY18, the chloroplasts were neatly arranged beside the cell walls and contained well-developed thylakoid membrane systems (Figures 3A,C). However, the cells of df74 showed plasmolysis, the number of chloroplasts was significantly less than that of NY18, and the chloroplasts were occupied by disordered thylakoids and 1–2 giant starch grains (Figures 3B,D).

### Inheritance of the Chl-Deficient Sepal Trait

The phenotypes of the generations obtained by the crosses of df74 and NY18 were thoroughly investigated. The reciprocal F_1_ plants exhibited green sepals as wild type, which indicated that the Chl-deficient sepal trait was controlled by nuclear genes. An F_2_ population with 322 individuals contained 239 green-sepal and 83 yellow-sepal plants. A chi-squared test indicated that the segregation pattern agreed with the Mendelian segregation ratio of 3:1 (χ^2^ = 0.067, *p* > 0.05). In addition, the ratio of green-sepal plants to yellow-sepal plants in the BC_1_ progenies was approximately 1:1 (χ^2^ = 0.019, *p* > 0.05). These results indicated that the phenotype of df74 was controlled by a single recessive nuclear gene.

### Bulked-Segregant Analysis With Whole-Genome Sequencing

**Figure 4:**
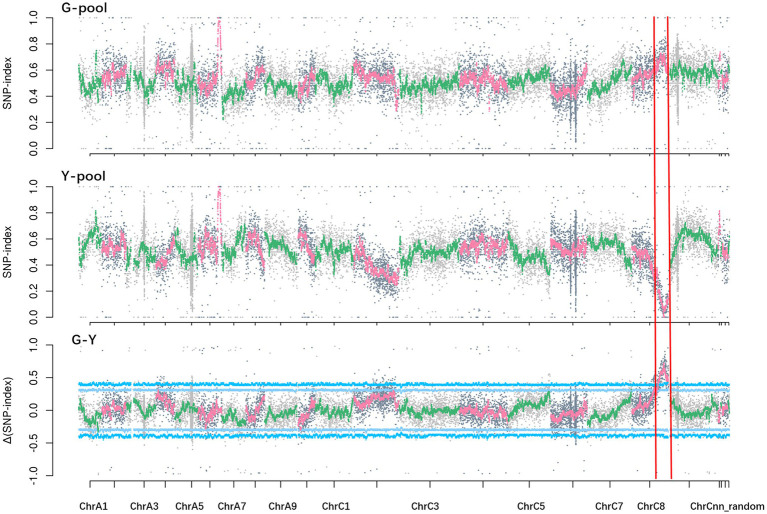
SNP-index (G-pool, Y-pool) and Δ(SNP-index; G-Y) graphs from the BSA-seq analysis. The *x* axes represent the *Brassica napus* chromosomes, and the *y* axes represent the SNP-index or Δ(SNP-index).

Solexa sequencing of the two pools and two parental lines generated about 172 Gb clean data, with Q20 ≥ 97.97% and Q30 ≥ 93.08%. The data were aligned to the *B. napus* reference genome “Darmor-*bzh*.” The average read depth was 39.61-fold in NY18, 26.77-fold in df74, 35.94-fold in the G-pool, and 36.89-fold in the Y-pool. A total of 26,900 differential and homozygous SNP loci were detected between two parental lines. At a 99% significance level, a single peak in the 26.22–37.54 Mb region on chromosome C08 had an average Δ(SNP-index) of 0.55. The largest Δ(SNP-index) among this genomic region was 0.81 (Figure 4). These data confirmed the presence of a major gene located in this genomic region controlling Chl-deficient sepal phenotype, which was designated as *BnC08.cds*.

**Figure 5:**
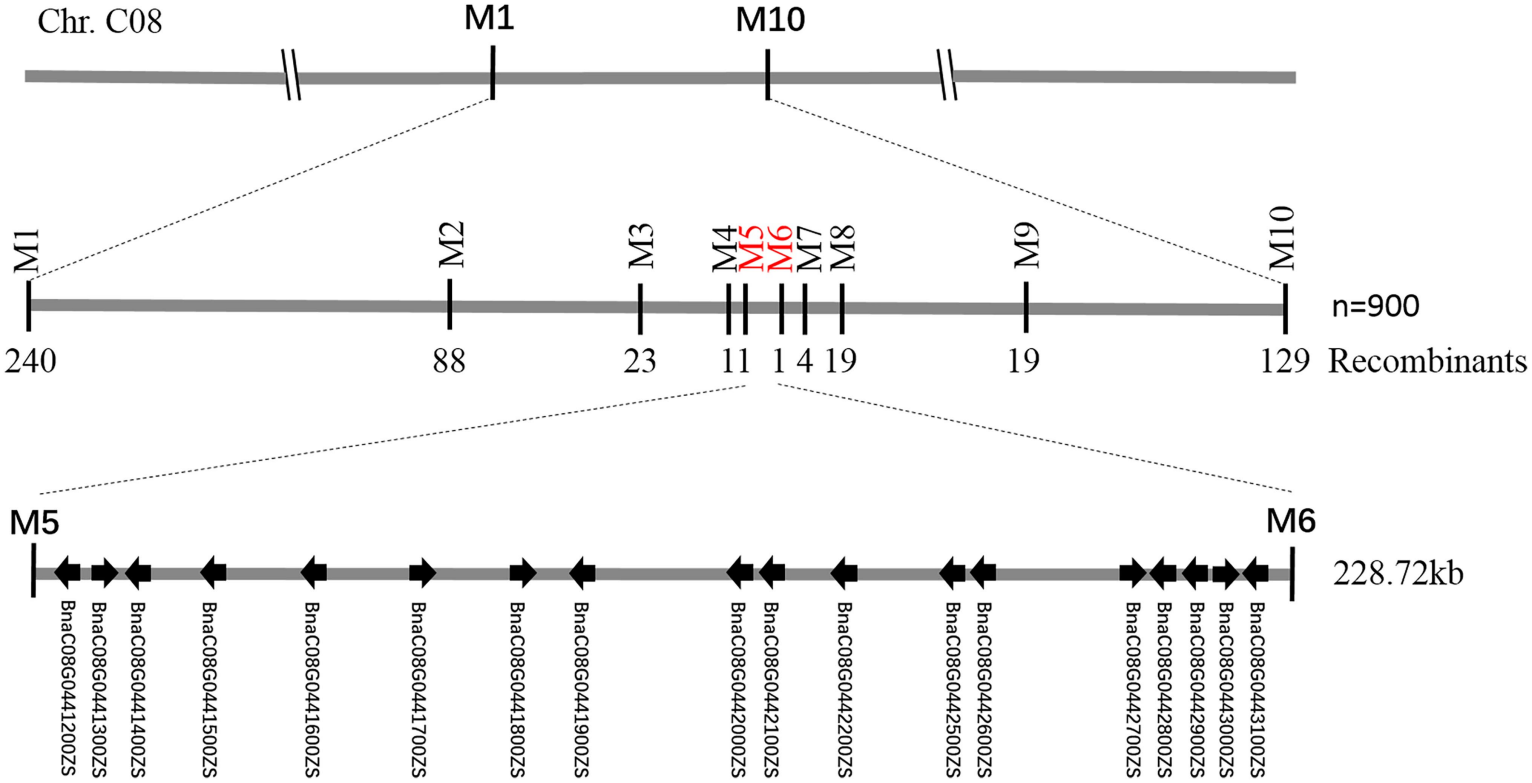
Fine-mapping of *BnC08.cds*. Using 900 recessive individuals of F_2_ population derived from the cross between df74 and ZS11, the *BnC08.cds* was mapped to a 228.72 kb interval on Chromosome C08, restricted by PARMS SNP markers M5 and M6.

### Fine-Mapping of the *BnC08.cds* Gene

To fine-map the *BnC08.cds* gene, the candidate region derived from BSA-seq was aligned to the reference genome sequence of *B. napus* “ZS11.” According to the result, two flanking PARMS SNP markers, M1 (physical position of the *B. napus* “ZS11” genome: 44420751) and M10 (50875750) were first designed to investigate the recombinants among the 900 yellow-sepal individuals in the (ZS11 × df74) F_2_ population. According to the result, 369 individuals containing recombinants between the two markers were selected. Furthermore, eight polymorphic PARMS SNP markers (M2–M9) were designed step by step according to the genome sequencing data (Table 1), and the recombinant individuals were analyzed. The *BnC08.cds* was finally mapped in an interval between M5 and M6 (Figure 5), both resulting in one recombinant. The region had a 228.72 kb physical distance and 18 candidate genes (Table 2) corresponding to the *B. napus* “ZS11” genome (Song et al., 2020).

**Table 1:** Penta-primer amplification refractory mutation system (PARMS) single-nucleotide polymorphism (SNP) markers used for fine-mapping of the *BnC08.cds* gene.

Marker name	SNP position	Primer	
		Name	Sequence
M1	44,420,751	M1-F	AGAAACTTAGAGCATGATTATCGGG
		M1-Rg	GAAGGTGACCAAGTTCATGCTCGTTTTCAATTTTTTTTTTCATCAG
		M1-Ra	GAAGGTCGGAGTCAACGGATTCGTTTTCAATTTTTTTTTTCATCAA
M2	46,664,419	M2-F	ATAAGATTTGACGAGGTTTGAATG
		M2-Rg	GAAGGTGACCAAGTTCATGCTTTCATAGATTTTAGAGAAATTAATATTCTG
		M2-Ra	GAAGGTCGGAGTCAACGGATTTTCATAGATTTTAGAGAAATTAATATTCTA
M3	47,651,192	M3-F	GACGGAATCGGATAATCGGAG
		M3-Rg	GAAGGTGACCAAGTTCATGCTCACGTACGCACCGCTAAATG
		M3-Ra	GAAGGTCGGAGTCAACGGATTCACGTACGCACCGCTAAATA
M4	48,137,207	M4-F	TTGTTTAGGGCTTTAGTTTTTGTGG
		M4-Rg	GAAGGTGACCAAGTTCATGCTCCAGTCAAGTATCAAGGCGTTGAG
		M4-Ra	GAAGGTCGGAGTCAACGGATTCCAGTCAAGTATCAAGGCGTTGAA
M5	48,165,304	M5-Fc	GAAGGTGACCAAGTTCATGCTTTTGTTGTAAGAAGAATTTGTTGGC
		M5-Ft	GAAGGTCGGAGTCAACGGATTTTTGTTGTAAGAAGAATTTGTTGGT
		M5-R	CACCATAGATTGACTTGGACACAGAC
M6	48,394,029	M6-Fc	GAAGGTGACCAAGTTCATGCTAATTATTTTCGTTTCATAATGTATACAAAC
		M6-Ft	GAAGGTCGGAGTCAACGGATTAATTATTTTCGTTTCATAATGTATACAAAT
		M6-R	ATTATATTTACTAAATAACCATCTTATGCTG
M7	48,444,442	M7-F	CTTTAGTGAGAATGTCAGCCTTTTG
		M7-Rc	GAAGGTGACCAAGTTCATGCTACGTAGAGCATATTGCAGGAAGC
		M7-Rt	GAAGGTCGGAGTCAACGGATTACGTAGAGCATATTGCAGGAAGT
M8	48,637,321	M8-Fc	GAAGGTGACCAAGTTCATGCTGTATGAATACAAATATGTAGTTATATAGAAAAAC
		M8-Ft	GAAGGTCGGAGTCAACGGATTGTATGAATACAAATATGTAGTTATATAGAAAAAT
		M8-R	AGAGTGATGAACGAAAAAAACAAG
M9	49,509,367	M9-Fc	GAAGGTGACCAAGTTCATGCTAAAAATTTTAGGCAATGATATCCTC
		M9-Ft	GAAGGTCGGAGTCAACGGATTAAAAATTTTAGGCAATGATATCCTT
		M9-R	AAAACCTAAAATGATGCAGCTCTAC
M10	50,875,750	M10-Fc	GAAGGTGACCAAGTTCATGCTCAGTTGAGAAAATCGGTCGAG
		M10-Ft	GAAGGTCGGAGTCAACGGATTCAGTTGAGAAAATCGGTCGAA
		M10-R	CAGAGATAAACTAATCAGACACAGACAG

**Table 2:** Genes on the mapped 228.72 kb interval of “ZS11” reference genome and their annotation.

Gene of *B. napus*	Chromosome position	Orthologous gene of *A. thaliana*	Gene function
BnaC08G0441200ZS	48,166,422–48,167,318	AT2G22790	Na
BnaC08G0441300ZS	48,172,282–48,173,172	AT3G43590	Zinc knuckle (CCHC-type) family protein
BnaC08G0441400ZS	48,178,061–48,180,109	CYP708A3	Cytochrome P450, family 708, subfamily A, and polypeptide 3
BnaC08G0441500ZS	48,193,353–48,194,143	FIB2	Fibrillarin 2
BnaC08G0441600ZS	48,214,869–48,215,139	HA2	H(+)-ATPase 2
BnaC08G0441700ZS	48,234,481–48,245,908	AT1G78500	Terpenoid cyclases family protein
BnaC08G0441800ZS	48,251,861–48,253,171	AT3G30280	HXXXD-type acyl-transferase family protein
BnaC08G0441900ZS	48,263,294–48,264,883	NAI1	Basic helix–loop–helix (bHLH) DNA-binding superfamily protein
BnaC08G0442000ZS	48,293,028–48,294,397	AT2G22760	Basic helix–loop–helix (bHLH) DNA-binding superfamily protein
BnaC08G0442100ZS	48,297,446–48,302,808	AT5G43745	Ion channel POLLUX-like protein, putative (DUF1012)
BnaC08G0442200ZS	48,312,997–48,314,566	AT2G22750	Basic helix–loop–helix (bHLH) DNA-binding superfamily protein
BnaC08G0442500ZS	48,334,374–48,337,660	AT2G22720	SPT2 chromatin protein
BnaC08G0442600ZS	48,340,523–48,341,329	BRK1	BRICK1, putative
BnaC08G0442700ZS	48,369,127–48,374,978	AT2G22620	Rhamnogalacturonate lyase family protein
BnaC08G0442800ZS	48,375,906–48,381,142	AT2G22610	Di-glucose binding protein with Kinesin motor domain
BnaC08G0442900ZS	48,382,029–48,385,287	AT2G22600	RNA-binding KH domain-containing protein
BnaC08G0443000ZS	48,387,732–48,388,852	NIC1	Nicotinamidase 1
BnaC08G0443100ZS	48,389,268–48,390,586	NET2D	Kinase interacting (KIP1-like) family protein

### Genome-Wide Transcriptomic Analysis of NY18 and df74

To further understand the molecular mechanisms of the difference between NY18 and df74, high-throughput RNA-seq was performed using Illumina technology. After the raw data were trimmed, 19.90–23.54 million clean reads for each sample were aligned to the *B. napus* “Darmor-*bzh*” genome. A total of 4,108 DEGs between NY18 and df74 were identified, including 1,550 genes upregulated and 2,558 downregulated in the sepals of df74. To investigate the function of the DEGs, GO enrichment analysis was performed. DEGs were mainly categorized and highly enriched as follows, “cellular process,” “metabolic process,” and “single-organism process” in the “Biological Process” category, and “binding” and “catalytic activity” in the “Molecular Function” category. The most abundant terms in the “Cellular Component” category were “cell,” “cell part,” “membrane,” “organelle,” and “membrane part” (Supplementary Additional File 1). Functional annotation of the DEGs was performed against the KEGG database to further investigate the functions of DEGs. The 1,522 up- and 2,527 downregulated DEGs were classified into 116 and 117 pathways, respectively. The result showed the most significantly enriched KEGG pathways were pentose and glucuronate interconversions, starch and sucrose metabolism, and metabolic pathways (Supplementary Additional File 2).

As color change is related to Chl content, we focused on the porphyrin and Chl metabolism pathways. Three DEGs that encode proteins participating in Chl breakdown were upregulated in df74, and one DEG participating in Chl biosynthesis, was downregulated. The three upregulated genes included *BnaA08g30360D*, *BnaC01g20010D*, and *BnaC01g01800D*. The first two genes were both homologous genes of *Methylesterase family member 16 (MES16)* in *Arabidopsis*, which catalyzes the demethylation of fluorescent Chl catabolite (Li and Pu, 2016). The third, *BnaC01g01800D*, was homologous to accelerated cell death gene *ACD2* in *Arabidopsis*, which encodes a Chl breakdown enzyme to protect cells from programmed cell death caused by porphyrin-related molecules (Pattanayak et al., 2012). The downregulated gene was *BnaC09g47630D*, which is the homologous gene of *HEMC* that encodes the porphobilinogen deaminase function in Chl biosynthesis (Lim et al., 1994). These pathway annotations of the DEGs revealed the functions of *BnC08.cds*.

### Identification of the Candidate Genes in the Mapped Interval

According to the fine-mapping results, the *BnC08.cds* was located in an interval with a 228.72 kb physical distance and 18 candidate genes (Table 2). The RNA-seq analysis indicated that none of the 18 genes had significantly different expression levels between the sepals of df74 and NY18. Six genes (*BnaC08G0441400ZS*, *BnaC08G0441600ZS*, *BnaC08G0441700ZS*, *BnaC08G0441800ZS*, *BnaC08G0442000ZS*, and *BnaC08G0442200ZS*) were not expressed in either df74 or NY18. These genes were excluded from the candidate genes.

In this interval, there was an enrichment region of basic helix–loop–helix (bHLH) DNA-binding superfamily genes, including *BnaC08G0441900ZS*, *BnaC08G0442000ZS*, and *BnaC08G0442200ZS*. The bHLH superfamily is one of the largest transcription factor gene families in *Arabidopsis* (Heim, 2003). Members in the phytochrome-interacting factor family that belongs to the bHLH superfamily VII can negatively regulate Chl biosynthesis (Huq et al., 2004; Shin et al., 2009). In view of this previous research, we cloned and aligned the sequence of the three genes with bHLH domain in df74 and NY18. There was no difference in the three genes between df74 and NY18; therefore, these genes were excluded as candidate genes for *BnC08.cds*.

*BnaC08G0441400ZS* is homologous to *CYP708A3* in *Arabidopsis*, which belongs to the cytochrome P450 gene family and functions in heme binding. We sequenced the *BnaC08G0441400ZS* in df74 and NY18. There is only one base mutation in the intron region, so it was also excluded as a candidate gene for *BnC08.cds*.

According to the result of resequencing, there was only one missense mutation in the 18 genes. In *BnaC08G0442100ZS*, there was a single nucleotide substitution (G–A) in the seventh exon, which changed serine into asparagine at the 232nd amino acid site. The mutation was confirmed by sequencing the gene in df74 and NY18. The orthologous gene of *BnaC08G0442100ZS* in *A. thaliana* is *AT5G43745*, which encodes an ion channel POLLUX-like protein and is located in the chloroplast and chloroplast envelope. Therefore, we speculated that *BnaC08G0442100ZS* may be the most likely candidate gene of *BnC08.cds*.

## Discussion

According to the pigment measurements, the Chl content of sepals in df74 was significantly lower than NY18. However, the fine-mapping revealed there was no missense mutant gene that directly participated in the biosynthesis and degradation of Chl or heme in the candidate region on chromosome C08. Therefore, the *BnC08.cds* might belong to the third category and function in chloroplast development. The factors involved in chloroplast development are numerous and varied. For example, the *YSA* gene in rice, which encodes a pentapeptide repetitive (PPR) protein, is required for chloroplast development in early seedling leaves (Su et al., 2012). The *ysa* mutant displays a stage-specific albino. Leaves of the mutant appear albino before the three-leaf stage, but gradually turn green from the four-leaf stage and return to normal green after the six-leaf stage. The PPR family is one of the largest families in plants. It functions in the regulation of gene expression in plastids, including transcription, splicing, RNA cleavage, RNA editing, translation, and RNA stabilization (Schmitz-Linneweber and Small, 2008). These kinds of PPRs involved in chloroplast development have been found in other species, including *DG1* (Chi et al., 2008) and *YS1* (Zhou et al., 2010) in *Arabidopsis*, and *PPR4* (Schmitz-Linneweber et al., 2006) and *PPR10* (Prikryl et al., 2011) in maize. As a tissue-specific albino, a tomato mutant *suffulta (su)* with albinic stems and visually normal leaves were collected (Chang et al., 2021). Map-based cloning showed that *Su* encodes a DnaJ heat shock protein, and the homolog gene *AtARC6* (accumulation and replication of chloroplasts) in *Arabidopsis* is involved in chloroplast division. Previous observations in *Arabidopsis arc6* mutants suggest that chloroplasts can divide during cell division (Marrison et al., 1999). By contrast, stem cells only undergo cell expansion, while leaf growth involves both cell division and cell expansion. These differences may lead to the tissue specificity of chloroplast development. In this study, the Chl-deficiency is sepal-specific. More research is needed to reveal the difference in chloroplast development between sepal and other green tissues.

Floral organs are believed to be modified versions of a ground-state organ similar to the leaf. The modifications are led by different combinations of MADS-domain transcription factors encoded by floral organ identity genes (Sablowski, 2015). The model system of flower development is well-established, but how the floral organ identity genes perform their functions and create the diverse morphology of floral organs is little known. Among the floral organs of most angiosperms, the sepals and stigmas have chloroplasts, while the petals and stamens rarely have. In petals and stamens, photosynthetic capacity is lost during modification. Researchers have speculated that the B-function genes suppress the differentiation of photosynthetic tissues. Two genes, *GNC* and *GNL*, which were implicated in the regulation of Chl biosynthesis, were both directly and negatively regulated by *AP3/PI* in petals and stamens (Mara and Irish, 2008). Another direct target of organ identity genes related to light responses is *BHLH161/BANQUO3* (*BNQ3*; Mara et al., 2012). The *bnq3* mutants have pale-green sepals and carpels and decreased Chl levels. The *BNQ3* is widely expressed in shoots but is directly repressed by *AP3/PI* in developing petals. Thus, one way in which B-function genes suppress the photosynthetic capacity in petals and stamens is by repressing regulatory genes involved in light response and chloroplast development. However, the photosynthetic capacity in sepals of most angiosperms persists during modification. This may have led to the assumption that the floral organ identity genes may not participate in the regulation of chloroplast development in sepals. But, in our study, the chlorosis in the mutant is sepal-specific. We speculate that the A-function genes may be involved in chloroplast development in sepals, and the *BnC08.cds* gene may be a target gene that regulated by floral organ identity genes, directly or indirectly.

The model system of flower development is well-established from the original ABC model (Coen and Meyerowitz, 1991) to the ABCE model (Theissen, 2001), new (A)BC model (Causier et al., 2010), and floral quartet model (Theißen et al., 2016). But how the floral organ identity genes perform their functions is little known. In recent years, it has become increasingly clear that the downstream genes of these master regulators execute many of the functions originally attributed to the floral organ identity factors (Thomson et al., 2017). Studies on the gene-regulatory networks and molecular mechanisms of reproductive development in plants have made tremendous progress in recent years (Wils and Kaufmann, 2017). Research on the downstream genes of the floral organ identity factors has become a focus of interest. The df74 with Chl-deficient sepals is valuable because this kind of sepal-specific chlorosis has rarely been reported. The *BnC08.cds* which controls the sepal-specific Chl-deficiency in df74 will be a good subject to study the regulation network of chloroplast development in sepals.

## Data Availability Statement

The datasets presented in this study can be found in online repositories. The names of the repository/repositories and accession number(s) can be found at: https://www.ncbi.nlm.nih.gov/bioproject/, PRJNA807748.

## Author Contributions

JZ and XW designed the research. WZ led and coordinated the overall research. FC, HZ, RS, CS, SC, and MH performed the research. WZ and XZ co-wrote the manuscript. XW revised the manuscript. All authors contributed to the article and approved the submitted version.

## Funding

The work was supported by National Natural Science Foundation of China (32001580), China Agriculture Research System of MOF and MARA (CARS-12), and Key Laboratory of Jiangsu Province for Agrobiology (JKLA2021-ZD02). All sources of funding received for the research were submitted.

## Conflict of Interest

The authors declare that the research was conducted in the absence of any commercial or financial relationships that could be construed as a potential conflict of interest.

## Publisher’s Note

All claims expressed in this article are solely those of the authors and do not necessarily represent those of their affiliated organizations, or those of the publisher, the editors and the reviewers. Any product that may be evaluated in this article, or claim that may be made by its manufacturer, is not guaranteed or endorsed by the publisher.

## Footnotes

^1^ https://www.genome.jp/kegg/

^2^ https://www.biostars.org/p/200126/

## Supplementary Material

Supplementary Additional File 1 Go classification of DEGs between sepals of NY18 and df74.

Supplementary Additional File 2 Pathway enrichment of DEGs between sepals of NY18 and df74.
