# Supplementary figures and images for: Fine Mapping and Candidate Gene Analysis of BnC08.cds, a Recessive Gene Responsible for Sepal-Specific Chlorophyll-Deficiency in Brassica napus L
Source: Front Plant Sci. 2022 Mar 10;13:850330. doi: 10.3389/fpls.2022.850330 (PMC8960310; doi:10.3389/fpls.2022.850330)

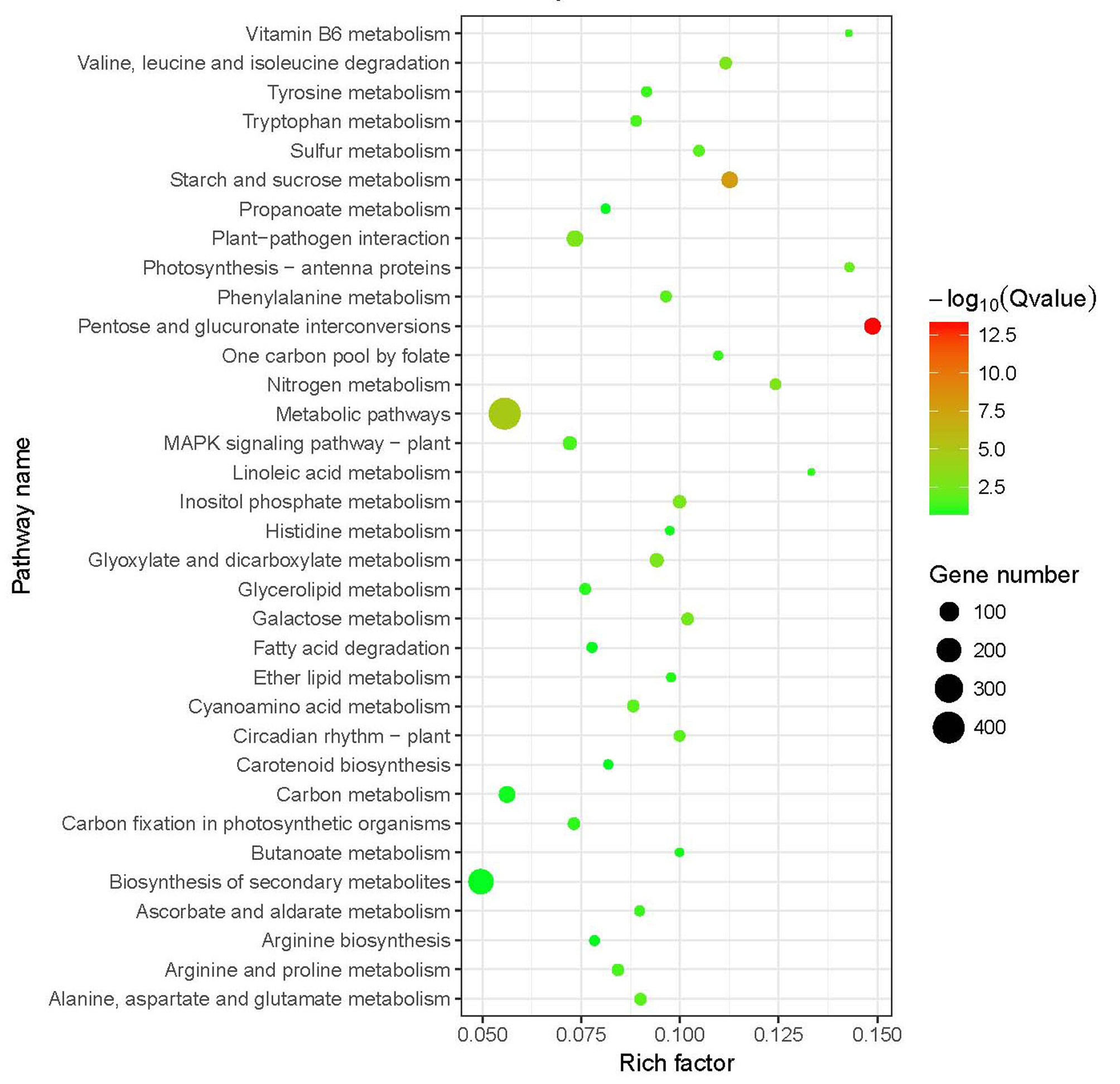

Supplement: Supplementary Additional File 2 — Pathway enrichment of DEGs between sepals of NY18 and df74. [file Image_1.TIF]
